# Empirically derived portion sizes from the DOrtmund Nutritional and Anthropometric Longitudinally Designed (DONALD) study for 4- to 18-year-old children and adolescents to simplify analysis of dietary data using FFQ

**DOI:** 10.1017/S136898002400017X

**Published:** 2024-01-23

**Authors:** Maike Elena Schnermann, Ute Nöthlings, Ute Alexy

**Affiliations:** Institute of Nutrition and Food Sciences, Nutritional Epidemiology, University of Bonn, Friedrich-Hirzebruch-Allee 7, 53115 Bonn, Germany

**Keywords:** Portion size, food intake, usual intake estimation, children, adolescents

## Abstract

**Objective:**

To apply FFQ, knowledge about portion sizes is relevant. According to increased energy and nutrient requirements, average portion sizes of foods are supposed to increase during growth. We provide empirically derived portion sizes for 4- to 18-year-olds in different age groups to facilitate analyses of FFQ data in children and adolescents.

**Design:**

Using data from the dynamic DOrtmund Nutritional and Anthropometric Longitudinally Designed cohort study, quantile regression for smoothing percentiles was used to derive portion sizes as a function of age from which age- and food group-specific portion sizes were calculated as median food group intake (g).

**Setting:**

Dortmund, Germany.

**Participants:**

Data from 3-day weighed dietary records (WDR) of 1,325 participants (♀: 653) were analysed. Participants provided in total 9,828 WDR (on average 7·5 per participant) between 1985 and 2022. WDR were grouped into five age groups, whereby each age group covered 3 years of age.

**Results:**

In total, 11 955 food items were reported and categorised into sixteen major food groups with seventy-one sub-groups. Portion sizes tended to increase with age, except for *milk*- and *plant-based alternatives*. Comparing 4- to 6-year-olds to 16- to 18-year-olds, portion size increased between 22·2 % (*processed meat*: 18 g *v*. 22 g) and 173·3 % (*savoury snacks*: 15 g *v*. 41 g).

**Conclusion:**

We provide empirically derived portion sizes for children and adolescents. These data are useful to establish dietary assessment methods based on estimates of portion sizes, such as FFQ, for children and adolescents.

Dietary assessment in general is challenging, but especially in children and adolescents, due to lower literacy skills and limited memory compared with adults^(1,2)^. Children’s food choices differ only slightly from those of adults, with the exception of age-appropriate adjustment of portion sizes^(3)^. Hence, dietary assessment methods similar to those used in adults, such as 24-h recall, dietary records or FFQ, are currently used to assess dietary intake in young populations^(4)^. In case of semi-quantitative tools like FFQ, researchers must apply information on portion sizes, the quantity of food consumed at one eating occasion, to quantify usual dietary intake. Unfortunately, there is a noticeable lack of reference values for portion sizes, especially tailored to different age groups in childhood and adolescence, highlighting a clear need for such data.

An FFQ is a commonly used survey method to retrospectively assess usual food intake over a past period of time, such as 6 or 12 months^(5)^. Participants are asked to indicate the frequency of intake (e.g. several times per day, per week and per month) of commonly consumed foods and beverages^(6)^. To quantify usual dietary intake and further calculate energy and nutrient intake, researchers need to multiply consumption frequencies by standard portion sizes. Although the list of foods included in the FFQ may be the same for children and adults, the portion sizes clearly differ. Therefore, knowledge of age-specific food portion sizes is crucial, especially in dietary assessment methods where participants are not encouraged to weigh their food and beverages, such as FFQ. For example, the FFQ used in the representative German National Health Interview and Examination Survey for Children and Adolescents (KiGGS) employed common household measures of forty-five food groups to estimate food intake. A validation study among 12- to 17-year-old adolescents concluded that the FFQ was suitable to rank children and adolescents based to their food intake^(7)^.

Age-appropriate food portion sizes are also required when dietary assessment is done by a 24-h recall. With this assessment method, participants retrospectively report the dietary intake over the past 24 h^(6)^. Given that children and adolescents have difficulties to estimate accurately their food and beverage consumption, researchers might provide time-consuming and labour-intensive tools, such as food photographs, household measures or two-dimensional grids for more precise assessments^(8)^. However, if detailed standard portion sizes were available for various age groups, it would greatly facilitate the post-processing of 24-h recalls in terms of both time and costs.

Up to now, empirically derived portion sizes for different age groups remain scarce for children and adolescents. Previously, only portion sizes of selected food groups for young children (6–36 months) were released as part of the German GRETA study^(9)^. The authors used data from the DOrtmund Nutritional and Anthropometric Longitudinally Designed (DONALD) study, a dynamic cohort study collecting weighed dietary records since 1985 from infancy to young adulthood^(10)^. With approximately 10 000 dietary records available, it is possible to calculate empirically age-specific portion sizes throughout childhood and adolescence. Therefore, the aim of the current analysis was to provide portion sizes for a wide range of food groups and age groups for children and adolescents aged 4–18 years.

## Research design and methods

### Study design

The DONALD study is an ongoing dynamic cohort study since 1985 in Dortmund, Germany. Its primary objective was to investigate the association between dietary intake, metabolism and growth from infancy into early adulthood. Details on recruitment, study design and methods have been published previously^(11)^. In brief, each year, 30–40 infants from the region of Dortmund were unsystematically recruited and follow-up into adulthood. Among the examinations are 3-d weighed dietary records (3dWDR), which were conducted annually from infancy onwards. The study is conducted according to the Declaration of Helsinki and was approved by the Ethics Committee of the University of Bonn. All examinations were carried out with written informed consent from study participants themselves (≥16 years) or their parents. The study was registered in the German Register of Clinical Trials (DRKS-ID: DRKS00029092).

### Study population

At the time of our data analysis, 10 880 3dWDR collected between 1985 and 2022 were available form 1,338 participants aged 4–18 years. Dietary records were categorised as underreported if the ratio of reported energy intake to estimated BMR according to the age- and sex-specific equations of Schofield^(12)^ was below the cut-off values proposed by Goldberg^(13)^. After exclusion of 921 (8·5 %) potential underreported dietary records, the final dataset consisted of 9,828 3dWDR from 1,325 participants (49·3 % female). To facilitate our analysis, we divided the dietary records into five groups based on the age of the participants: 4–6 years, 7–9 years, 10–12 years, 13–15 years and 16–18 years. Due to the nature of our open and on-going cohort study, the number of dietary records as well as the number of participants gradually decreased with increasing age groups. It is important to note that participants in the first age group (4–6 years) may not have yet reached the age range for the next age group (7–9 years) and will therefore have the opportunity to submit dietary records within that age group in the coming years.

### 3-day weighed dietary records

Parents or older participants themselves collected detailed information on all foods and beverages consumed over three consecutive days using electronic food scale (±1 g). Since data on dietary intake in younger children (<7 years) are often considered less reliable^(1)^, but dietary recording already starts in infancy, it is carried out by parents. Since then, participating families conduct annual dietary records and with increasing age and cognitive abilities, children assist by recording guided by their families who have routine in weighing and recording of foods through years of practice. Findings from the DONALD study indicated that 80 % of participants aged 10–12 years assisted their parents in recording their dietary intake. By the time they reached the age of 15–18 years, 70 % of the boys and 90 % of the girls were able to independently maintain their dietary records without parental assistance^(14)^. When participants consumed food away from home, such as at school or in a restaurant, and were unable to weigh all consumed items, they were permitted to provide semi-quantitative estimates such as household measurements or the number of portions. Participants were requested to document generic food items as accurately as possible, including details such as the fat content of milk or the type of grain (whole grain or refined grain) in dishes. For commercially packaged food items, participants collected both the packages and the accompanying food labels. Nutrient information and ingredient lists from each newly recorded food item were used to simulate recipes, enabling the addition of commercially available food items to the in-house food composition table LEBTAB, resulting in a continuously growing food database^(15)^.

### Food group classification

Classification of foods and beverages is a complex task, as there is no standardised procedure for classification into food groups available. To derive portion sizes, we have therefore used three steps: First, the 11 570 food items from the LEBTAB database of the DONALD study were categorised based on shared characteristics (e.g. milk: milk, chocolate milk and strawberry milk) or commonly culinary usage (e.g. cream: cream, sour cream, crème fraiche). Second, we aggregated food groups according to those usually used in FFQ and 24-h recall. In case of composite dishes, the large heterogeneity and diversity of the products made it necessary to aggregate them into joint groups. Third, we took the grouping of the German Food Book into account, to be as consistent as possible with the eating habits^(16)^. Two researchers collaboratively defined the major food groups and corresponding underlying food groups. One scientist assigned all items to the previously defined food groups. In case of ambiguity, a second scientist was consulted for clarification. A total of 2,247 food items were excluded for the analysis as separate portion sizes for these groups were deemed unnecessary for estimating usual food intake. Those included commercial infant or toddler foods (e.g. baby mixed dishes or milk-cereal mixtures for infants, *n* 236), instant powder (e.g. for sauces, *n* 546), supplements (e.g. vitamins or minerals, *n* 334), flour and baking mixtures (e.g. baking mixes for bread or cakes, *n* 205), alcoholic beverages (e.g. beer, wine or whisky, *n* 49) and herbs and spices (e.g. salt, garlic granules or dried basil, *n* 35). It also included food items that could not be assigned to any broader food group, such as additives, colourants or preservatives (*n* 842). The remaining 9,323 items were then classified into sixteen major food groups: dairy, eggs, meat, fish, fat/oil, cereals, potatoes, vegetables, fruits, pulses, beverages, sweets, snacks, spreads, dishes and plant-based alternatives.

To ensure precision in portion size determination, these sixteen major food groups were further subdivided into a total of seventy-one underlying food groups. This subdivision allowed us to segregate foods that exhibited substantial differences in typical consumption quantities. For instance, the food group *dairy* was subdivided into *milk, fermented dairy, cheese, curd cheese and cream*. A comprehensive list of the major food groups, along with their respective underlying food groups and descriptions of associated food items, can be found in see online supplementary material, S1 Table.

### Additional covariables

Trained nurses assessed participants’ weight (kg) and height (m) to calculate their BMI (kg/m^2^). Further, the LMS method was used to determine the BMI sd score (SDS), using age- and sex-specific values for median (M), CV (S) and skewness (L) from the German national reference^(17)^.

### Statistical analysis

All statistical analyses were conducted using the statistical software SAS (Version 9·4). Participants’ characteristics were presented as median values (25th percentile; 75th percentile) for continuous variables and as relative frequencies (%) for categorical variables unless otherwise noted.

To derive median individual portion sizes based on actual consumption, we exclusively included participants who consumed quantities greater than 0 g within the respective food group. Since portion sizes are strongly dependent on age, it is useful to smooth the percentiles. By using a quantile regression (PROC HPQUANTSELECT), random fluctuations in the percentiles were eliminated effectively^(18)^. This approach allowed us to represent portion sizes as a simple function of age. The rationale behind this procedure is that minor changes in the covariate age are likely to result in continuous alterations in portion size, thus changing uniformly across different age groups^(19)^. Subsequently, we calculated the median amount (in g) of weighed food group per eating occasion for each food group, which represents the empirically derived portion size for this respective age group. Differences in food portion sizes between boys and girls were tested with the Mann–Whitney *U* test to identify the need to report sex-specific portion sizes. We did observe sex differences in food portion sizes for a small number of underlying food groups, namely milk, vegetable juices and energy drinks (*P* < 0·05). However, these differences were not consistent across the age groups. Thus, portion sizes for children (4–12 years) were not stratified by sex. Due to significant differences in energy intake among adolescents, we decided to present sex-specific portion sizes for adolescents (13–18 years).

## Results

Participant characteristics are described in Table 1. The number of participants as well as the number of 3dWDR decreased continuously between the youngest age group (4–6 years, *n* 2,847) and the oldest age group (16–18 years, *n* 1,113). In contrast, total energy intake increased by a median of 68 % from the youngest to the oldest age group (1,331 *v*. 2,239 kcal). With regard to the overall study population, the median age was 9·9 years (25th percentile: 6·2; 75th percentile: 13·0) and 7·5 dietary records (range: 1–16) were on average available per participant.

**Table 1:** Characteristics* of 1,325 study participants of the DONALD study

	4–6 years		7–9 years		10–12 years		13–15 years		16–18 years	
Number of participants	1,143		989		843		652		534	
Number of 3dWDR	2,740		2,437		2,034		1,432		1,110	
Age (years)	5·1	4·4; 6·0	8·1	7·3; 9·0	11·1	10·4; 12·0	14·1	13·2; 15·0	17·1	16·2; 18·1
Female participants	565	49·4	471	47·6	416	49·4	312	47·9	266	49·8
Total energy intake (kcal/d)^†^	1,331	1·177; 1,510	1,633	1,442; 1,835	1,858	1,625; 2,094	2,098	1,793; 2,431	2,239	1,885; 2,671
Girls	1,267	1,132; 1,431	1,544	1,366; 1,724	1,718	1,525; 1,969	1,871	1,649; 2,111	1,891	1,686; 2,137
Boys	1,401	1,238; 1,582	1,718	1,533; 1,920	1,953	1,749; 2,200	2,326	2,061; 2,678	2,630	2,312; 2,936
Number of 3dWDR per participant within age group	3	2; 3	3	2; 3	3	2; 3	2	2; 3	2	1; 3
n_3dWDR_ per participant and age group										
1	185	16·2	141	14·3	114	13·5	158	24·2	158	29·6
2	319	27·9	248	25·1	267	31·7	208	31·9	176	33·0
3	639	55·9	600	60·6	462	54·8	286	43·9	200	37·4
BMI SDS	0·03	–0·49; 0·57	−0·10	–0·64; 0·56	−0·08	–0·72; 0·61	−0·01	–0·72; 0·62	0·01	–0·69; 0·67
Girls	0·04	–0·48; 0·59	−0·10	-0·64; 0·57	−0·09	–0·73; 0·66	−0·01	–0·79; 0·62	0	–0·71; 0·69
Boys	0·03	–0·50; 0·54	−0·10	–0·64; 0·54	−0·05	–0·71; 0·57	0	–0·66; 0·63	0·04	–0·66; 0·65

3dWDR, 3-day weighed dietary records; SDS, Standard Deviation Score.

* Data shown as median (25th percentile; 75th percentile) or relative frequency (%).

† Calculation of total energy intake includes all foods and beverages consumed, not just those used for portion size derivation.

Reported frequencies within each food and age group (see online supplementary material, S2 Table) ranged from no consumption (e.g. *wraps*, 4–6 years) to 19 582 times (*bread*, 4–6 years). In case of no reported consumption, it was not possible to derive portion sizes for the respective age groups, which was the case for *energy drinks* in three age groups, *instant dishes* in one age group, *ready-to-eat salad with sauce* in two age groups, *premade grain-based salad* in four age groups and *Traditional German dishes* in one age group. Similar cases were identified for certain underlying food groups within the major food group *plant-based alternatives*. Across all food groups, the median of reported frequencies was 482 (25th percentile: 39; 75th percentile: 2,278). In terms of reported frequencies, 49·9 % of the underlying food groups had reported frequencies below the median, mostly covering *plant-based alternatives* and *dishes*.

The empirically derived portion sizes (Table 2) cover all food groups except for *plant-based alternatives*. Minimum portion size was 2 g (*liquid oil* or *solid fat*, 4–6 years) and maximum portion size was 500 g (*energy drink*, for 16–18-year-old boys). In general, portion sizes increased with age across all food groups, except for the food group *milk* and all food groups belonging to the major food group *plant-based alternatives*. When comparing the youngest with the oldest age group, it is noticeable that portion size increases between 22·2 % (*processed meat*: 18 g *v*. 22 g) and 286·7 % (*savoury snacks*: 15 g *v*. 58 g) for general food groups. Regarding *dishes*, portion sizes increased between 38·1 % (*soup*: 194 g *v*. 268 g) and 1,190·3 % (*Traditional German Dishes*: 31 g *v*. 400 g). Portion sizes for the major food group *plant-based alternatives*, including 12 underlying food groups, are provided in the appendix (see online supplementary material, S3 Table).

**Table 2:** Empirically derived portion sizes in gram for 4–18-year-old children and adolescents from 9,828 3-d weighed records

		Portion size (g) per eating occasion						
Major food group	Underlying food group	4–6 years	7–9 years	10–12 years	13–15 years (boys)	13–15 years (girls)	16–18 years (boys)	16–18 years (girls)
Number of participants		1,143	989	843	340	312	268	266
Number of 3dWDR		2,740	2,437	2,034	686	746	553	557
Dairy	Milk	130	156	175	205	152	218	150
	Fermented dairy	106	125	130	150	138	150	150
	Cheese	15	18	20	23	20	25	21
	Curd cheese	28	34	36	40	38	50	38
	Cream	11	15	17	20	20	24	24
Eggs	Eggs	16	17	17	20	18	28	24
Meat	Beef/pork	35	48	55	78	66	88	66
	Poultry	40	56	65	94	75	108	77
	Processed meat	18	19	20	21	20	22	20
	Meat dishes	63	75	88	111	100	120	107
Fish	Fish	40	44	52	67	53	67	64
	Fish dishes	70	80	99	128	99	130	99
Fat/oil	Liquid oils	2	3	3	4	4	5	4
	Margarine/butter	4	5	5	6	6	6	6
	Frying oil	3	3	5	6	4	6	4
	Solid fat	2	3	3	3	3	3	3
Cereals	Bread	31	37	41	46	44	49	44
	Rice (cooked)	60	78	96	115	115	139	122
	RTEC, muesli, flakes	24	32	41	55	43	69	49
	Pasta (cooked)	89	120	135	182	140	200	161
Potatoes	Potatoes	70	88	104	138	123	160	127
	Potato dishes	74	97	108	136	120	140	120
Vegetables	Vegetables	70	90	102	126	109	126	109
	Leafy vegetables (raw)	16	21	25	29	29	29	29
	Juice	93	111	129	136	124	136	226
Fruits	Fruits	65	75	82	93	93	96	100
	Fruit puree	92	100	107	140	123	162	130
	Juice	86	102	125	170	164	200	180
	Dried fruits	10	10	10	10	15	10	18
	Nuts	5	6	7	8	8	14	8
Pulses	Pulses (cooked)	39	50	57	70	56	76	65
Beverages	Water based	118	150	200	242	210	283	242
	Sugar sweetened	158	200	200	294	224	322	250
	Energy drinks	n/a	n/a	n/a	250	250	500	370
Sweets	Candies	10	10	12	20	11	21	14
	Chocolate	14	17	20	24	20	25	21
	Ice cream	48	56	62	70	68	80	70
	Cake	51	60	60	70	62	85	68
	Cream cakes	72	93	99	101	110	110	111
	Cookies	18	20	25	39	28	40	32
	Flour dishes	60	60	60	60	60	78	60
	Desserts	122	125	137	146	138	159	150
Snacks	Savoury snacks	15	19	30	41	34	58	35
Spreads	Spreads	10	12	15	17	15	18	15
Dishes	Pizza	100	150	191	269	200	330	241
	Gratin	150	200	256	400	267	467	270
	Bread with meat	103	113	118	209	120	209	150
	Kebab	134	200	354	400	370	400	380
	Soup	194	240	217	290	267	377	268
	Pasta dishes	181	199	299	327	283	376	286
	Wraps	n/a	93	115	150	170	197	169
	Instant dishes	n/a	223	235	235	247	248	250
	Rice dishes	64	68	150	210	122	227	183
	Spring rolls	58	88	97	130	125	152	147
	Premade salad with mayo	54	73	80	140	110	190	142
	Premade salad with dressing	n/a	n/a	195	199	n/a	280	300
	Premade grain-based salad	n/a	n/a	n/a	n/a	n/a	232	79
	Pan-fried dishes	100	137	261	n/a	245	395	250
	Traditional German dishes	31	84	220	n/a	n/a	400	227

n/a, not available; RTEC, ready-to-eat-cereals.

Consumption frequencies ≥482 (median of reported frequencies across all underlying food groups) were marked in light grey, and consumption frequencies <482 were marked in white. Of note, the database of vegetarian and vegan products covers a relatively small number of food items (n 237). In addition, the reported frequencies were below the median (482), and thus portion sizes might therefore not be considered as highly reliable.

## Discussion

Using the comprehensive data from the DONALD study, we have empirically determined portion sizes for sixteen major food groups, encompassing a total of seventy-one underlying food groups. These determinations have been made across five distinct age groups spanning from 4 and 18 years and additionally for both sexes from the age of 13 years. As expected, our analyses have shown a consistent increase in portion weights with increasing age. It is important to note that the values represents actual portion sizes observed within our study sample and do not constitute evidence-based intake recommendations.

Our empirically derived portion sizes align closely with those reported by Wrieden and colleagues. Their portion sizes were drawn from the British National Diet and Nutrition Survey, where dietary intake was similarly assessed through the use of weighed food records^(20)^. They chose age ranges that are directly comparable to those in our analysis and derived portion sizes from an extensive dataset comprising 3,374 weighed dietary records encompassing 1,675 children and adolescents. Given that Wrieden and colleagues analysis is more than 15 years old, a new derivation of portion sizes might be beneficial, as portion sizes may have evolved over time or new food groups may have emerged. While some distinctions in food group categorization exist between the DONALD study and the British population, the resulting portion sizes are notably similar. For instance, in the case of pasta, for individuals aged 16–18 years, our study indicates a portion size of 161–200 g, whereas the British dataset suggests 185 g. Similarly, for milk consumption among 7- to 9-year-olds, our findings indicate 156 g, while the British dataset suggests 186 g. Of note, the British portion sizes are always slightly larger than the German portion sizes. Various factors affect portion sizes, including energy intake or energy requirement^(21)^, dietary habits or cultural background^(22)^. Unfortunately, Wrieden and colleagues did not provide specific information on energy intake, thus precluding an assessment of its comparability to the DONALD participants. Likewise, no information was available concerning energy requirements based on the level of physical activity.

The German Nutrition Society (DGE) has established portion size guidelines as part of its quality standards for catering in kindergartens^(23)^ and schools^(24)^, ranging from 1 to 19 years. Although portion sizes derived from the DONALD study align closely with the DGE’s recommendations, our portion sizes often are below those recommended by the DGE. For rice or potatoes, the DGE recommends 90 g for children aged 4–6 years, whereas the portion sizes observed in the DONALD study were 60 and 70 g, respectively. We found similar results for legumes among 7–9-year-olds (DGE: 80 g *v*. DONALD: 50 g). Of note, portion sizes of the DGE serve as intake recommendations to reach the recommended nutrient intakes, whereas portion sizes of the DONALD study reflect actual consumption amounts. In addition, it is not clear on which data the DGE portion sizes are based or whether they were derived empirically at all.

While weighed dietary records are considered as the gold standard in dietary assessment and generally represent precise tools^(25)^, there are certain limitations, which potentially introduce inaccuracies in portion sizes determination. First, the prospective nature of these records can lead to underreporting of unhealthy foods and overreporting of healthy foods^(26)^. Consequently, this can led to the reporting of a dietary pattern, which is socially acceptable but may not truly represent the usual diet. Second, portions sizes reported for unhealthy foods and beverages may be consciously or unconsciously underestimated due to social desirable bias^(27)^, although we excluded records with underreported energy intake for more reliable portion sizes. Third, accurately estimating food consumption away from home presents unique challenges, given the variability in portion sizes and ingredients^(28)^. Notably, only one percent of the records was mainly based on semi-quantitative estimates, ensuring that our portion sizes reflect real-world consumption. Overall, these issues are particularly pertinent among younger children, where surrogate reporting is often necessary due to limited self-reporting capabilities^(1)^.

Research on sex-specific portion sizes for children and adolescents is scarce. Considering sex-specific portion sizes may increase the accuracy of consumption assessments for absolute nutrient intakes in adults^(29–31)^. Analyses in our sample suggest that sex-specific portion sizes are not necessary until adolescence, as consumption levels are very similar before puberty, although energy intake is higher in boys at all ages. Obviously, the higher energy intake is not reflected in the portion size of each food group. From late adolescence, when physiological differences as well as changes in body composition between girls and boys are more prominent, sex-specific portion sizes should be taken into account, which is why we calculated sex-specific portion sizes from 13 years onwards. Of note, due to sex stratification, the number of reported food items in some food groups is been considerably lower due to their infrequent consumption in our study sample and should be used with caution.

The major food group *plant-based alternatives*, which is a novel food group emerging due to the trend towards plant-based vegetarian and vegan diets^(32)^, should be discussed in particular. The market for products, such as soya, rice, almond or oat drinks as milk alternatives, or plant-based alternatives for fish, meat and eggs, is steadily growing^(33)^. Hence, portion sizes for these products are also desirable, primarily to facilitate the development of dietary assessment instruments for vegetarians and vegans. Furthermore, given that omnivores increasingly incorporate these alternatives into their diets, the relevance of portion size information extends beyond plant-based eaters. With the predominant omnivore study sample of the DONALD study, these products have only been consumed in significant frequencies since 2016. This has resulted first in a limited number of available food items and second in consumption frequencies per food item below the median of reported frequencies within our study sample. However, even now, our data indicate lower portion sizes for alternative plant-based products compared with their animal source counterparts (16–18 years for *milk v*. *milk alternatives:* 150–218 g *v*. 96–127 g).

It is plausible that changes in dietary habits may not result in a proportionate increase in portion sizes in line with energy requirements. A reduction in milk consumption during late adolescence is well documented^(34,35)^. We observed similar findings in our data. Despite statistical adaptation, consumption of *milk* was larger in girls aged 13–15 years than in girls aged between 16 and 18 years (152 g *v*. 150 g). One possible explanation for this shift could be that milk is no longer consumed in its pure form beyond the age of 16 but is instead used as an additive for coffee or ready-to-eat-cereals. Our data reveal an increase in *vegetables* with age. Nevertheless, this increase might primarily stem from a higher nutrient demand rather ran an increased energy demand, as vegetables typically contribute minimally to total energy intake due to their low energetic content. The reason for this age-related rise in vegetable consumption could be attributed to the enhanced sensory acceptance of vegetable taste, texture and smell^(36)^.

In the German KiGGS study, food consumption data obtained from the validated FFQ were transformed into mean intakes as grams per day using predefined portion sizes^(7)^. Age ranges in the KiGGs study are considerably broader than in our study, making direct comparisons challenging due to differences in age grouping. However, when comparing specific portion sizes, such as desserts (DONALD 142–159 g *v*. KiGGS: 150 g), there is a notable degree of concordance. In contrast, portion sizes for chocolate (DONALD: 14–25 g *v*. KiGGS: 50 g) or nuts (DONALD: 7–14 g *v*. KiGGS: 25 g) show less agreement. In general, it appears that the portion sizes reported in the KiGGS study are slightly larger than those observed in the DONALD study. Of note, dietary intake in g of the 1,249 participants from the KiGGS study was roughly estimated, while in the DONALD study dietary intake was actually weighed.

Definition of age groups and categorisation of food groups might be essential for portion sizes. Our classification was based on subjective judgement, although we made diligent efforts to document our decisions (see online supplementary material, S1 Table) to ensure replicability. Food grouping is a complex task and during the process of coding, it might be necessary to revise initial decisions. For instance, we initially subdivided vegetable oils into three food groups. The first group encompassed rapeseed and sunflower oil, which are commonly used in Germany for frying. The second group included olive oil, primarily used for salad dressings and thus typically in smaller quantities. The last group included expensive oils, such as nut oils, linseed oil or soybean oil, used in smaller quantities due to their cost and culinary value. However, our statistical analyses showed that the consumption levels across all age groups were identical or differed only slightly (±1 g). Therefore, we combined all vegetable oils to increase the number of consumption occasions, resulting in more reliable portion size estimates.

Strengths and limitations of the used data should be mentioned. The study sample is characterised by a high socio-economic status, which limits the generalisability of our findings to the broader German population. Participants with a low socio-economic status are difficult to recruit due to the study’s long-term and time-consuming design^(10)^. Consequently, participants with very unhealthy dietary habits might not be adequately represented in our dataset. In addition, the study is geographically limited to the region of Dortmund and thus regional differences in dietary intake cannot be represented. If more northern areas with seaside access were included, consumption of fish and fish products might have been more frequent, providing more robust estimates. Furthermore, we observed only a few food groups where portion sizes did not increase with age, such as *rice dishes* (10–12 years: 150 g *v*. 13- to 15-year-old girls: 122 g). This might be due to the small number of recorded rice dishes (*n* 7) among 13- to 15-year-old girls. Despite the high number of 9,828 available weighed dietary records, reported frequencies for certain food groups might be too low to calculate reliable portion sizes, necessitating caution when using these estimates. The strength of the study is the multiple collected dietary data with a mean of 7·5 3dWDR per participant. In addition, weighted dietary records often serve as reference instruments for dietary assessment methods. Nevertheless, when weighing is not possible (i.e. out of home consumption), participants are allowed to use household measurements. However, in only 5·8 % of the dietary records, between 50 and 75 % of all items consumed were reported using household measurements.

In conclusion, our current analysis provided empirically derived age-specific food portion sizes for a wide range of food groups spanning 4 and 18 years. They are also likely to be particularly useful for evaluating 24-h recalls or FFQ in young age groups. Nevertheless, there is a compelling need for further research to validate our portion sizes in other populations.

## Supporting information

Schnermann et al. supplementary material Schnermann et al. supplementary material

## Supplementary material

For supplementary material accompanying this paper visit https://doi.org/10.1017/S136898002400017X
