# Supplementary material for: Empirically derived portion sizes from the DOrtmund Nutritional and Anthropometric Longitudinally Designed (DONALD) study for 4- to 18-year-old children and adolescents to simplify analysis of dietary data using FFQ
Source: Public Health Nutr. 2024 Jan 23;27(1):e49. doi: 10.1017/S136898002400017X (PMC10882525; doi:10.1017/S136898002400017X)
Supplement: Schnermann et al. supplementary material [file S136898002400017Xsup001.docx]

# Supplemental material for “Empirically derived portion sizes from the DONALD study for 4 to 18 year old children and adolescents to simplify analysis of dietary data using FFQ”

**S1 Table:** Definition of food groups

| **Major food group (n items)** | **Underlying food group (n items)** | **Description** |
| --- | --- | --- |
| Dairy (1,070) | Milk (106) | Milk, flavoured milk, latte macchiato & coffee with milk (refrigerated) |
|  | Fermented dairy (611) | Yogurt, drinking yogurt, soured milk, kefir, buttermilk, probiotic yoghurt drinks, skyr |
|  | Cheese (240) | Cream cheese, soft cheese, sliced cheese, processed cheese, sandwich slices, feta, mozzarella, grated cheese, parmesan, cottage cheese, mascarpone, ricotta |
|  | Curd cheese (72) | Curd cheese, curd cheese with herbs, aioli, sour cream, tzatziki |
|  | Cream (41) | Cream, sour cream, crème fraiche |
| Eggs (18) | Eggs (18) | Whole egg, scrambled egg, fried egg, omelette, egg salad |
| Meat (811) | Beef/Pork (64) | Beef, pork, veal, lamb, game, goat: as fillet, roast, minced meat, steak |
|  | Poultry (26) | Chicken, duck, turkey, goose, ostrich |
|  | Processed meat (529) | Meat salad, salami, mortadella, meat sausage, liver sausage, cold cuts |
|  | Meat dishes (192) | Meatballs, fillet strips, stuffed turkey breast, escalope, shashlik, gyros, fricassee, goulash, ragout, roulades, meatballs, cordon bleu |
| Fish (218) | Fish (54) | Fish, canned fish, smoked fish, seafood, shellfish |
|  | Fish dishes (164) | Fish salads, ready meals, breaded fish |
| Fat/Oil (316) | Liquid oils (48) | Rapeseed oil, alba oil, olive oil, sunflower seed oil, corn germ oil, wheat germ oil, palm oil, vegetable oil, linseed oil, walnut oil, soybean oil, peanut oil, almond oil, hazelnut oil, pumpkin seed oil, hemp seed oil, borage oil, enriched oils, algae oil, safflower oil, grape seed oil, oat oil, cottonseed oil |
|  | Margarine/Butter (225) | Butter, butter variations (herb butter, garlic butter), margarine, vegan spreads, butter blends (butter with rapeseed oil) |
|  | Frying oil (36) | Oil used for food preparation, e.g. Rama |
|  | Solid fat (7) | Frying fat, palm fat, coconut fat, palm kernel fat, peanut butter, cocoa butter, shea butter |
| Cereals (1,795) | Bread (929) | Bread, toast, rolls, pretzels, flatbread, crispbread, bagel, focaccia, baguette, raisin bread, yeast bread, brioche, sticks, sweet rolls, croissants |
|  | Rice (cooked) (27) | Rice, amaranth, bulgur, quinoa, pearl barley, millet |
|  | RTEC, muesli, flakes (717) | Whole-grain breakfast cereal, muesli, cornflakes, other refined grain-based breakfast cereal |
|  | Pasta (cooked) (122) | Cereal pasta, lentil pasta, stuffed pasta |

| **Major food group (n items)** | **Underlying food group (n items)** | **Description** |
| --- | --- | --- |
| Potatoes (123) | Potatoes (41) | Salted potato, jacket potato, mashed potato (without powder), roasted potato, dumpling (also bread dumpling) |
|  | Potato dishes (82) | French fries, gnocchi, potato noodles, croquettes, duchess potatoes, hash browns, potato pockets (frozen), potato wedges, potato salad |
| Vegetables (208) | Vegetables (278) | All fresh, frozen and dried vegetables except leafy vegetables, creamed vegetables, buttered vegetables, vegetable mixes |
|  | Leafy vegetables (raw) (12) | Dandelion, salad, spinach, chard, arugula, sorrel, purslane, amaranth leaves, baobab leaves |
|  | Juice (18) | Carrot juice, tomato juice, beet juice, spinach juice, pumpkin juice |
| Fruits (601) | Fruits (90) | All fresh, frozen and canned fruits |
|  | Fruit puree (131) | Puree, Smoothies |
|  | Juice (301) | 100% fruit juice, juice spritzers, multivitamin juice |
|  | Dried fruits (30) | Raisins, dates, figs, acai berry and all other dried fruits |
|  | Nuts (49) | Nuts and seeds |
| Pulses (27) | Pulses (cooked) (27) | Beans, lentils, peas, tofu, tempeh |
| Drinks (924) | water-based (354) | Mineral water, drinking water, flavoured water, tea, coffee |
|  | sugar-sweetened (559) | Cola, Fanta, Sprite, isotonic drink, nectar, draught soda, iced tea, tonic, including artificially sweetened beverages |
|  | Energy drinks (11) | e.g. Red Bull, Monster |
| Sweets (1,886) | Candies (220) | Chewing candies, dextrose, gummy bears |
|  | Chocolate (494) | Chocolate, candy bars, pralines, cereal bars |
|  | Ice cream (281) | Ice cream, water ice |
|  | Cake (202) | Cakes, fruit cakes, doughnuts, cream puffs, éclairs, quark balls, strudels, chocolate soufflés |
|  | Cream cakes (71) | Cakes with cream, custard/cream, ricotta cream or mascarpone, cheesecake |
|  | Cookies (372) | Cookies, rusks, spoon cookies, rice cakes, gingerbread |
|  | Flour dishes (26) | Crepes, pancakes, waffles |
|  | Desserts (220) | Semolina pudding, rice pudding, custard, tiramisu, chocolate mousse, tartufo, crème brulee, jelly, red fruit jelly |

| **Major food group (n items)** | **Underlying food group (n items)** | **Description** |
| --- | --- | --- |
| Snacks (182) | Savoury snacks (182) | Potato chips, tortilla chips, crackers, salt sticks, grissini, flips, salted nuts |
| Spreads (270) | Spreads (270) | Jam, chocolate cream, vegetable spreads |
| Dishes (537) | Pizza (200) | Pizza, pizza baguette, tarte flambée, quiche, Stuffed puff pastry with spinach or cheese |
|  | Gratin (23) | Potato gratin, baked pasta dishes, vegetable gratin |
|  | Bread with meat (32) | Burgers (meat, fish, vegetables) with toppings, hot dogs, meatball rolls |
|  | Kebab (7) | Kebab with meat or vegetables, Kebab meat with fried potatoes |
|  | Soup (94) | Vegetable or potato soup, meat or fish soup, broth, thickened soup |
|  | Pasta dishes (74) | Stuffed pasta with sauce, pasta dishes (frozen), instant dishes |
|  | Wraps (8) | Ready-made wraps with meat or vegetables |
|  | Instant dishes (12) | Instant dishes based on rice, potatoes or noodles |
|  | Rice dishes (31) | Rice with vegetables, meat or fish, e.g. paella, nasi goreng |
|  | Spring rolls (9) | Spring rolls with meat or vegetables |
|  | Premade salad with mayo | Potato salad, pasta salad, vegetable (e.g. coleslaw), chicken salad, meat salad, egg salad |
|  | Premade salad with dressing | Ready-made leaf salad mixes with toppings and salad dressing |
|  | Premade grain-based salad | Bulgur, Couscous, Quinoa |
|  | Pan-fried dishes (8) | Roast potatoes with chicken, cereal pans (millet, quinoa, bulgur, couscous, spelt), chicken pans |
|  | Traditional German dishes (5) | Cabbage rolls, green cabbage with kassler, savoy cabbage rolls, kassler with sauerkraut |

| **Major food group (n items)** | **Underlying food group (n items)** | **Description** |
| --- | --- | --- |
| Plant-based alternatives (237) | Milk alternatives (65) | Drinks from almond, coconut, oat, soy, rice, cashew, spelt, hemp, hazelnut, buckwheat drink, millet drink, quinoa, lupine |
|  | Fermented milk alternatives (18) | Soy yogurt, coconut yogurt, oat yogurt, soy skyr, lupine yogurt |
|  | Cheese alternatives (6) | Cream cheese, sliced cheese, mozzarella cheese |
|  | Curd cheese alternatives (1) | Soy curd |
|  | Cream alternatives (11) | Soy cream, rice cream, oat cream, coconut cream |
|  | Egg alternatives (1) |  |
|  | Meat alternatives (7) | Saitan, minced meat, sliced meat, fillet |
|  | Cold cut alternatives (28) | Liver sausage, mortadella, ham pickerel, cold cuts, salami, meat salad, meat sausage |
|  | Meat dish alternatives (92) | Sausages, fillets, meatballs, burger patties, nuggets, escalope, bratwurst |
|  | Fish dish alternatives (1) | Fish sticks |
|  | Dessert alternatives (7) | Dessert alternatives from soy, hemp, lupines, rice |

RTEC: ready-to-eat-cereals

**S2 Table: Total reported frequencies over 3 days of all food groups**

| **Major food group** | | **Underlying food group** | **4-6 years** | **7-9 years** | **10-12 years** | **13-15 years (boys)** | **13-15 years (girls)** | **16-18 years (boys)** | **16-18 years (girls)** |
| --- | --- | --- | --- | --- | --- | --- | --- | --- | --- |
| Dairy | | Milk | 12,890 | 11,009 | 7,740 | 2,933 | 2,381 | 2,047 | 1,739 |
|  |  | Fermented dairy | 3,880 | 3,095 | 2,379 | 950 | 801 | 588 | 596 |
|  |  | Cheese | 6,433 | 6,298 | 5,680 | 2,208 | 2,311 | 1,966 | 2,075 |
|  |  | Curd cheese | 1,099 | 1,056 | 1,000 | 385 | 371 | 304 | 234 |
|  |  | Cream | 2,215 | 2,129 | 1,902 | 709 | 692 | 597 | 559 |
| Eggs | | Eggs | 4,079 | 3,685 | 2,899 | 1,097 | 1,045 | 758 | 702 |
| Meat | | Beef/Pork | 2,487 | 2,437 | 2,229 | 864 | 730 | 707 | 534 |
|  |  | Poultry | 997 | 959 | 824 | 332 | 281 | 285 | 248 |
|  |  | Processed meat | 10,340 | 9,745 | 7,874 | 3,134 | 2,370 | 2,486 | 1,887 |
|  |  | Meat dishes | 340 | 350 | 309 | 127 | 106 | 124 | 119 |
| Fish | | Fish | 753 | 716 | 622 | 256 | 238 | 253 | 206 |
|  |  | Fish dishes | 615 | 562 | 335 | 128 | 120 | 94 | 74 |
| Fat/Oil | | Liquid oils | 4,082 | 3,970 | 3,545 | 1,446 | 1,306 | 1,131 | 1,055 |
|  |  | Margarine/Butter | 15,269 | 13,604 | 10,379 | 4,005 | 3,415 | 3,047 | 2,613 |
|  |  | Frying oil | 217 | 189 | 159 | 83 | 69 | 44 | 71 |
|  |  | Solid fat | 269 | 243 | 217 | 55 | 80 | 58 | 59 |
| Cereals | | Bread | 19,582 | 18,606 | 15,106 | 5,855 | 5,074 | 4,692 | 4,118 |
|  |  | Rice (cooked) | 1,150 | 1,026 | 880 | 377 | 342 | 260 | 288 |
|  |  | RTEC, muesli, flakes | 3,235 | 2,988 | 2,136 | 796 | 616 | 571 | 487 |
|  |  | Pasta (cooked) | 2,453 | 2,205 | 1,715 | 653 | 642 | 571 | 531 |
| Potatoes | | Potatoes | 4,063 | 3,447 | 2,633 | 908 | 801 | 663 | 584 |
|  |  | Potato dishes | 728 | 780 | 636 | 212 | 231 | 179 | 181 |
| Vegetables | | Vegetables | 17,714 | 16,421 | 13,054 | 4,766 | 4,884 | 3,832 | 4,078 |
|  |  | Leafy vegetables (raw) | 933 | 1,128 | 1,134 | 442 | 558 | 380 | 489 |
|  |  | Juice | 135 | 78 | 51 | 27 | 12 | 20 | 15 |
| Fruits | | Fruits | 12,990 | 10,640 | 7,599 | 2,516 | 2,717 | 1,732 | 2,185 |
|  |  | Fruit puree | 493 | 364 | 267 | 101 | 101 | 60 | 57 |
|  |  | Juice | 12,099 | 9,631 | 6,979 | 2,560 | 2,213 | 1,828 | 1,573 |
|  |  | Dried fruits | 568 | 379 | 303 | 110 | 75 | 87 | 85 |
|  |  | Nuts | 2,278 | 1,933 | 1,514 | 557 | 586 | 395 | 384 |
| **Major food group** | | **Underlying food group** | **4-6 years** | **7-9 years** | **10-12 years** | **13-15 years (boys)** | **13-15 years (girls)** | **16-18 years (boys)** | **16-18 years (girls)** |
| Pulses | | Pulses | 544 | 492 | 399 | 163 | 115 | 105 | 127 |
| Drinks | | water-based | 24,150 | 20,530 | 16,828 | 6,255 | 6,357 | 5,536 | 6,118 |
|  |  | sugar-sweetened | 4,298 | 5,166 | 4,965 | 2,377 | 1,881 | 1,798 | 1,468 |
|  |  | Energy drinks | *n/a* | *n/a* | *n/a* | 10 | 2 | 18 | 12 |
| Sweets | | Candies | 4,696 | 4,331 | 2,642 | 628 | 671 | 236 | 376 |
|  |  | Chocolate | 5,404 | 5,303 | 4,371 | 1,493 | 1,401 | 802 | 975 |
|  |  | Ice cream | 2,562 | 2,375 | 1,744 | 485 | 486 | 249 | 254 |
|  |  | Cake | 1,446 | 1,304 | 1,015 | 336 | 325 | 208 | 227 |
|  |  | Cream cakes | 194 | 193 | 150 | 73 | 60 | 64 | 44 |
|  |  | Cookies | 2,683 | 2,128 | 1,377 | 379 | 429 | 234 | 306 |
|  |  | Flour dishes | 369 | 281 | 182 | 62 | 47 | 31 | 44 |
|  |  | Desserts | 824 | 763 | 588 | 280 | 202 | 180 | 160 |
| Snacks | | Savoury snacks | 1,113 | 1,150 | 1,086 | 363 | 350 | 205 | 222 |
| Spreads | | Spreads | 6,677 | 5,837 | 4,448 | 1,679 | 1,422 | 1,255 | 1,110 |
| Dishes | Pizza | | 285 | 356 | 401 | 175 | 146 | 175 | 108 |
|  | Gratin | | 27 | 21 | 18 | 7 | 11 | 6 | 7 |
|  | Bread with meat | | 38 | 59 | 82 | 43 | 45 | 73 | 42 |
|  | Kebab | | 6 | 13 | 35 | 22 | 14 | 40 | 17 |
|  | Soup | | 156 | 100 | 73 | 33 | 30 | 22 | 22 |
|  | Pasta dishes | | 52 | 56 | 52 | 30 | 23 | 21 | 22 |
|  | Wraps | | *n/a* | 2 | 2 | 3 | 1 | 4 | 4 |
|  | Instant dishes | | *n/a* | 2 | 6 | 3 | 8 | 6 | 5 |
|  | Rice dishes | | 14 | 17 | 11 | 1 | 7 | 6 | 6 |
|  | Spring rolls | | 13 | 14 | 18 | 11 | 9 | 3 | 6 |
|  | Premade salad with mayo | | 29 | 33 | 32 | 11 | 14 | 17 | 11 |
|  | Premade salad with dressing | | *n/a* | *n/a* | 4 | 2 | n/a | 1 | 1 |
|  | Premade grain-based salad | | *n/a* | *n/a* | *n/a* | n/a | n/a | 4 | 1 |
|  | Pan-fried dishes | | 1 | 2 | 1 | n/a | 4 | 3 | 1 |
|  | Traditional German dishes | | 1 | 2 | 3 | n/a | n/a | 2 | 1 |

| **Major food group** | **Underlying food group** | **4-6 years** | **7-9 years** | **10-12 years** | **13-15 years (boys)** | **13-15 years (girls)** | **16-18 years (boys)** | **16-18 years (girls)** |
| --- | --- | --- | --- | --- | --- | --- | --- | --- |
| Plant-based alternatives | Milk alternatives | 176 | 77 | 85 | 36 | 31 | 35 | 59 |
|  | Fermented milk alternatives | 28 | 17 | 6 | 4 | 1 | 3 | 8 |
|  | Cheese alternatives | 6 | *n/a* | *n/a* | 2 | 1 | 1 | *n/a* |
|  | Curd cheese alternatives | *n/a* | *n/a* | *n/a* | *n/a* | 1 | *n/a* | *n/a* |
|  | Cream alternatives | 25 | 20 | 21 | 9 | 15 | 14 | 19 |
|  | Egg alternatives | *n/a* | *n/a* | 4 | *n/a* | 1 | *n/a* | *n/a* |
|  | Meat alternatives | 3 | 3 | 3 | 1 | 1 | 2 | 1 |
|  | Cold cut alternatives | 45 | 34 | 39 | 15 | 21 | 8 | 30 |
|  | Meat dish alternatives | 48 | 35 | 39 | 17 | 30 | 15 | 33 |
|  | Fish dish alternatives | *n/a* | *n/a* | *n/a* | *n/a* | *n/a* | *n/a* | 1 |
|  | Dessert alternatives | 5 | 3 | 5 | 7 | *n/a* | *n/a* | 1 |

n/a: not available, RTEC: ready-to-eat-cereals

**S3 Table:** Empirically derived portion sizes in g for 4 to 18 year old children and adolescents from weighed records for plant-based alternatives

|  |  | Portion size (g) per eating occasion | | | | |  |  |
| --- | --- | --- | --- | --- | --- | --- | --- | --- |
| **Major food group** | **Underlying food group** | **4-6 years** | **7-9 years** | **10-12 years** | **13-15 years (boys)** | **13-15 years (girls)** | **16-18 years (boys)** | **16-18 years (girls** |
| Plant-based alternatives | Milk alternatives | 96 | 61 | 71 | 112 | 100 | 127 | 96 |
|  | Fermented milk alternatives | 97 | 51 | 77 | 73 | 50 | 101 | 152 |
|  | Cheese alternatives | 13 | *n/a* | *n/a* | 17 | 20 | 24 | *n/a* |
|  | Curd cheese alternatives | *n/a* | *n/a* | *n/a* | 17 | 8 | 24 | *n/a* |
|  | Cream alternatives | 17 | 21 | 20 | 37 | 12 | 50 | 29 |
|  | Egg alternatives | *n/a* | *n/a* | 1 | *n/a* | 3 | *n/a* | *n/a* |
|  | Meat alternatives | 10 | 16 | 51 | 62 | 8 | 16 | 10 |
|  | Cold cut alternatives | 11 | 13 | 12 | 24 | 12 | 23 | 15 |
|  | Meat dish alternatives | 40 | 57 | 64 | 90 | 80 | 54 | 63 |
|  | Fish alternatives | *n/a* | *n/a* | *n/a* | 90 | 80 | 54 | 63 |
|  | Fish dish alternatives | *n/a* | *n/a* | *n/a* | 90 | *n/a* | 54 | 87 |
|  | Dessert alternatives | 125 | 116 | 125 | 125 | *n/a* | 16 | 120 |

All consumption frequencies were <482.

n/a: not available
